# Communication in a medical setting: can standards be improved?

**DOI:** 10.1186/2049-6958-8-1

**Published:** 2013-01-07

**Authors:** Silvia Rossi Ferrario, George Cremona

**Affiliations:** 1 Psychology Unit, ‘S. Maugeri’ Foundation, IRCCS, Scientific Institute of Veruno (NO), Veruno, Italy; 2 Servizio di Pneumologia e Fisiopatologia Respiratoria, IRCCS. Ospedale S. Raffaele, Milan, Italy

**Keywords:** Communication, Medical setting, Skills

## Abstract

Do standards exist to improve communication in a medical setting? What are the minimal requirements to make our communication with patients and their family clear and simple? International literature, as well as psychology, philosophy, and even our brain structure offer ways to improve communication. We reflected about what is preventing effective communication in the medical setting and how/from where should we set about improving it.

## Review

Do standards exist to improve communication in a medical setting? Or, more to the point, what are the minimal requirements to make our communication with patients and their family clear and simple? International literature, as well as psychology, philosophy, and even our brain structure offer ways to improve communication. From this viewpoint, we discuss the 'real' question, that is what is preventing effective communication in the medical setting and how/from where should we set about improving it.

### What do we know about good communication in the medical setting?

#### International literature

**Figure 1:**
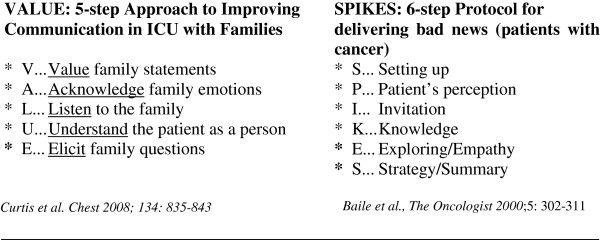
Approaches to communication with family and patients: VALUE and SPIKES.

Papers are continuously published in international medical journals about communication. The primary starting point is to clarify the object of communication. We often think about “how to communicate bad news”, but we have only a partial idea about what bad news is for a patient and his/her family. The majority of the papers addressing this issue refer mainly to advanced diseases or end of life. Fallowfield & Jenkins, [1] define bad news as “any information that produces a negative alteration to a person’s expectations about their present and future”. This means that, even if a gradation in bad news exists, what is important is the subjective perception of the patient which in turn depends on individual history and life experience, personality, beliefs, social support and any other human characteristics. In this sense, communicating to a patient (and his/her family) an end of life prognosis is not more difficult or less important that communicating to another the need of long term oxygen therapy which will cause the loss of his/her job, or the need to take on a very complex pharmacological therapy or limited life style. So, the starting point is that communication is an important event, independently from that we have to communicate, as its object will change the persons’ life to some extent. In other words, communicating is a therapeutic action [2]. The importance of subjective perception is too often underestimated. We know that every person has a 'cognitive elaboration' of the information received (i.e. a comprehension of the words' meaning in linguistic terms) and an 'affective elaboration' (i.e. the emotional resonance of the spoken word and the meaning which it acquires in terms of subjective quality of life, personal values, expectations and desires) [3,4]. Cognitive and affective elaborations often travel on different trajectories and on different time scales, often surprising health professionals on hearing that a patient or a caregiver is able to repeat exactly what has been said while giving an unexpected interpretation to the information. Health professionals often underestimate patients' need of information while overestimating their comprehension and awareness of the prognosis [5]. This seems also true for caregivers, who may perceive the patients' needs and difficulties differently from the health professionals as well as from the patients themselves [6-8]. A number of important characteristics regarding the context and the verbal and non-verbal behavior have been summarized by Ptacek & Eberhardt in 1996 [9], successively updated, and also presented in form of guidelines [10]. All authors agree that communication between doctor and patient should take place in an adequate context, preferably a private room, without interruption by people or phone, and with adequate time dedicated to it. It is not unusual that some communication takes place in corridors, where busy doctors are chased by the patient and family members, or in the bedroom in the presence of other sick persons. Careful attention has to be posed on non-verbal communication, such as eye contact (if culturally appropriate), open body position, active listening showed by head signs. To offer an emblematic example of what we are discussing, consider the dialogue in the W. Allen comedy (from “Play it again Sam”, 1972), "What are you doing on Saturday evening?”, “I'm busy, I'm going to commit suicide” “Oh, what about Friday evening?”. Behavioral strategies are suggested, starting from eliciting a person's understanding [11]. For instance, some authors have observed that many adults patients with a limited health literacy have difficulty understanding and remembering information given by their physician [12]. This underlines the importance of a more effective communication achieved by taking into consideration the patients’ ability to comprehension. Emphasis on the expected functional state and quality of life as well as emotional support have been shown to be the real need of patient and family [13-15]. Sastre et al. [16]. assert that support cannot be fully compensated by high quality of information, which is the contrary to the thesis popular among physicians for whom such a compensation is possible. Involvement of the patient and family in the decision making process with respect of their cultural and religious beliefs [17] is preferable. The use of empathic statements as well as increasing the proportion of time spent listening rather than talking has been advised [18]. Acronyms have been proposed to facilitate health professionals in remembering the steps to take as in the case of VALUE [18] and SPIKES [19]**(**Figure 1**)**.

In both approaches attention is focused on the patient with his/her particular way of perceiving the situation, his/her emotion and need to be aided in the decisional process by the physician’s empathic understanding.

#### Philosophy

The importance given by international literature to the individual is not new: about 2,500 years ago Plato, who lived in Athens between 427 and 347 BC., citing Socrates, said that “the real problem is not what we discuss about, but who is talking” [20]. Similarly, Hippocrates’s famous statement that: “Life is brief, art (craft) is long, opportunity evanescent, experience misleading, judgment difficult. The physician must not only be prepared to do what is right himself, but also to make the patient, the attendants, and externals cooperate” implies the importance of concentrating all resources to care of the individual. In a modern way of interpreting this affirmation, the attendants may be the members of the team and the family; the externals may be referred to organizational problems inside and outside the hospital, as, for example, the difficulties in the continuity of adequate care at home.

#### Brain structure

Our brain structure is itself an instrument of empathy [21-23]. Recent neuroimaging studies have recognized that specific brain areas are involved in decoding physical and emotional pain. These areas are known as “Pain Matrix”, although there is some discussion regarding the suitability of this name. The Pain Matrix is activated not only when we feel actual physical pain (which has emotional components) but also when we see other people suffering from it. Studies have shown that the more one is empathic the more the Pain Matrix is activated.

**Figure 2:**
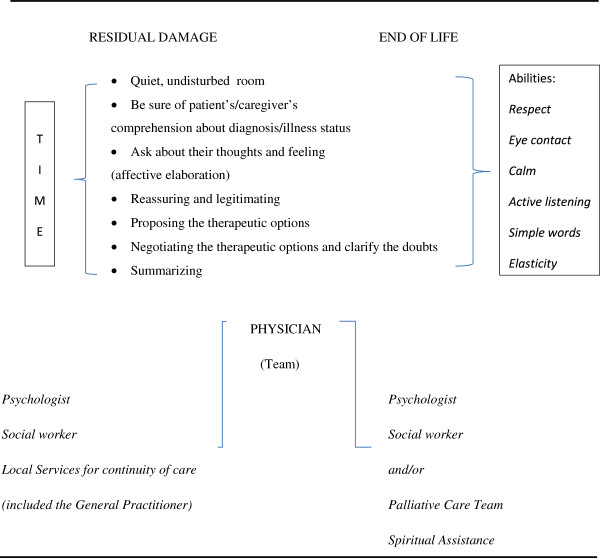
**Summary of the minimal requirements for a better medical communication.** *Careful medical communication is always important: time dedicated, abilities involved, context and physician’s/team’s attitudes should receive the same attention.

Considering all of these points, we can observe that we know and potentially have the minimal requirements to communicate better. This is summarized in Figure 2, considering that residual damage of different grade of severity and terminal disease should receive the same attention by health professionals.

The real question is then: why are we not able to apply these minimal requirements? What obstructs their application?

### Barriers to adequate communication

One of the most reported obstacles to adequate communication by health professionals, particularly physicians, is “lack of time”. In fact, health professionals are in general pressured by a lot of activities, many of which are bureaucratic. At the same time, they have to care for a considerable number of patients and, consequently, they have to relate to a potentially large number of caregivers, in a process of unbalanced expectations. Furthermore, conflicts between team members are not rare. Care-team members are rarely able to choose each other: they very often meet together for a case and, even when they choose to work in a specific area, people who meet may have opinions, points of view, motivations, as well as personal characteristics which differ very much from theirs. Azoulay et al. [24], noted that personal animosity, mistrust, lack of regular staff meetings, misunderstanding and lack of leadership were some of the most probable causes of intra-teams conflicts. Psychological defenses also may constitute other barriers in communicating bad news. In 1984, Buckman [25] listed a number of physicians’ fears potentially responsible for lack of communication with the patients: fear of being blamed, fear of the unknown and untaught, fear of unleashing a reaction, fear of expressing emotion, fear of not knowing all the answers, personal fear of illness and death. Personal history and personality’s characteristics may account for these fears and consequent psychological defenses and need of particular attention. In fact, professional formation in communication is necessary, but not sufficient in maintaining the improvements over time [1].

## Conclusions

Based on the evidence presented above, we suggest a number of practical steps to improve communication.

1. Self and intra-team evaluation could be the first step in clarifying the issues to focus on and identify those which may require further investigation. A number of manuals regarding evaluation are available [26,27] and may offer a method. Training courses or even, sometimes, personal psychotherapy are very often suitable.

2. Narrative medicine is another step helping towards the appraisal of one’s emotions and especially to be able to think of patients as persons with their own life history and not to forget that they are people before being patients. A common failing among health practitioners is to consider the patient only in the context of his disease as if there was nothing else prior to the illness. Contrary to what is we are often led to believe, defining patients in terms of their personal and unique context is a key element in engaging them and helping in the way that is important to them [28].

3. Modern philosophy can stimulate concrete changes. Hannah Arendt in 1958 [29] wrote: “What I propose in the following is a reconsideration of the human condition from the vantage point of our newest experiences and our most recent fears. This obviously, is a matter of thought, and thoughtlessness – the heedless recklessness or hopeless confusion or complacent repetition of truths which have become trivial and empty – seems to me among the outstanding characteristics of our time. What I propose, therefore, is very simple: it is nothing more than to think of what we are doing”. How can we translate this apparently simple suggestion in our daily work? A useful start would be to pay attention to the words that we use when talking about a patient and his/her caregiver: “he/she is the one in bed 1”, “he/she is a terminal patient”, “he/she is in a vegetative state”, he/she is the relative of bed 2”, “he/she is a pain in the neck”, he/she is someone who does not accept the situation” are some of the most common sentences we usually may say and hear. These sentences create a distance in some way from the concept of *person,* legitimizing a less careful behavior. Becoming aware of this mechanism activates our process of change. Awareness is a not an obvious and simple condition. It means living permanently in the present, alert and attentive to the present, without falling into the trap of preference or aversion, wishes and refusals, habits, emotions and unchallenged destructive thinking patterns whatever the importance of the matter at hand or however high the goal [30]. Learning moment-to-moment awareness reduces inadequate communication and negative emotions, as one is aware of what should be communicated, of the fact that he/she is communicating, of his/her way to communicate. In the same time, he/she is aware of what another person is communicating to him/her, of the fact that this person is not merely the specific behavior in that moment as well as which emotions are being expressed. We become able to observe and listen to the person communicating with us with respect and attention, and aware of our own attitude. From a practical point of view, as it is unlikely that we will be able to dedicate enough time to all of our patients/caregivers, we have to select those who need particular attention, ‘avoiding to avoid’ the more difficult ones. The coordinators (of nurses, physicians and every other involved figure) should decide some organized meetings where the health professionals involved exchange opinions about the patients' management problems and personal difficulties. This does not exclude that everyone should learn to be sufficiently elastic and available when it is needed to speak each other urgently. In relation to the specific moment of communication with the patient/caregiver (but not only!), we should prepare ourselves before the meeting, taking some minutes to reflect, observing our emotions, our prejudices, our projective attitudes, our expectations. This can help us to reduce the mistakes during communication and to maintain our concentration on the objective. If we cannot do this, it is always helpful to take some minutes after the meeting, in order to think about what and how happened and, if necessary, to rectify the situation.

In conclusion, we feel that the necessity of focusing on the barriers that impede an adequate communication and therefore preclude an optimal physician-patient relationship is obvious. It is equally evident that it is possible to identify a starting point from which to reduce these barriers. The current socio-economic situation and the inevitable need of rationing the healthcare the world over should not constitute a reason to avoid improvement.

As stated by Gallagher and Levinson [31]_**,**_ interactions between anxious patients and frustrated physicians are a prescription for conflict and non-adherence. A number of papers have underlined the correlation between a good doctor-patient relationship and patient’s health improvement [32]_**,**_ effectiveness of the care [33]_**,**_ and a reduction of utilization of care facilities [14]_**,**_ as well as a reduction of length of hospital stay and admission to institutional care in presence of a coordinated multidisciplinary team [34]_**.**_ Similarly, others have exhorted health professionals to protect their relationship with their patients even in the light of the current economic situation and with the aim of a greater efficiency in care [35,36]_**.**_ Following the tradition of acronyms, we should like to propose ESTATE indicating the heritage which has to be accumulated with experience and never lost: E, elect patient/caregiver (who should I select), S, share with the team, T, think about what I am going to communicate, A, act, T, track the patient/caregiver and observe their behavior and attitude, E, evidence what it has worked and what has to be done again.

## Competing interest

The authors declare that they have no competing interests.
